# Supplementary material for: Front-biased activation of the Ras-Rab5-Rac1 loop coordinates collective cell migration
Source: J Cell Sci. 2025 Aug 4;138(15):jcs263779. doi: 10.1242/jcs.263779 (PMC12377714; doi:10.1242/jcs.263779)
Supplement: Supplementary information [file joces-138-263779-s1.pdf]

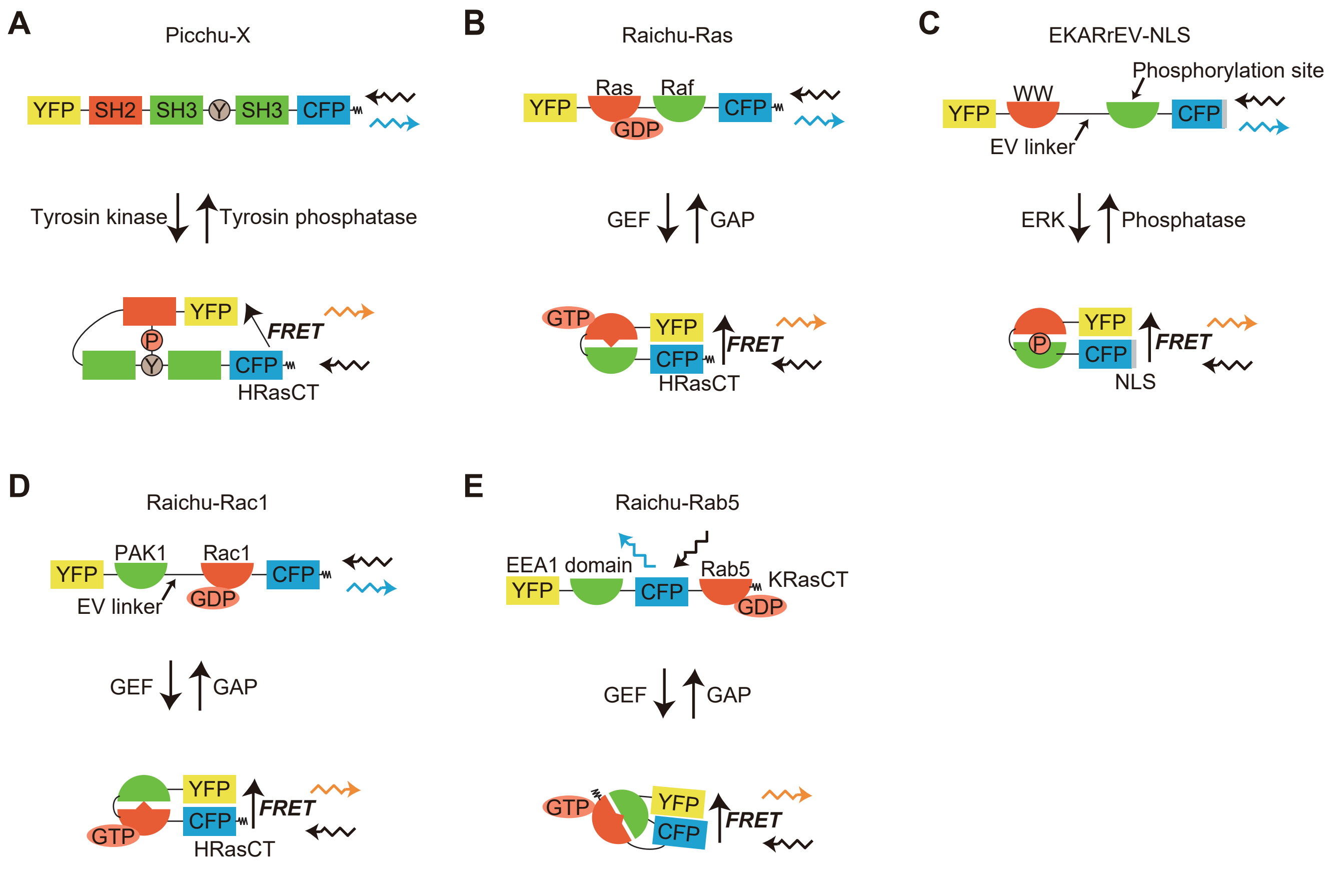

Fig. S1. Schematics of FRET biosensors

(**A-E**) Schematics of FRET biosensors, Picchu-X (A), Raichu-Ras (B), EKARrEV-NLS (C), Raichu-Rac1 (D), or Raichu-Rab5 (E).

Journal of Cell Science • Supplementary information

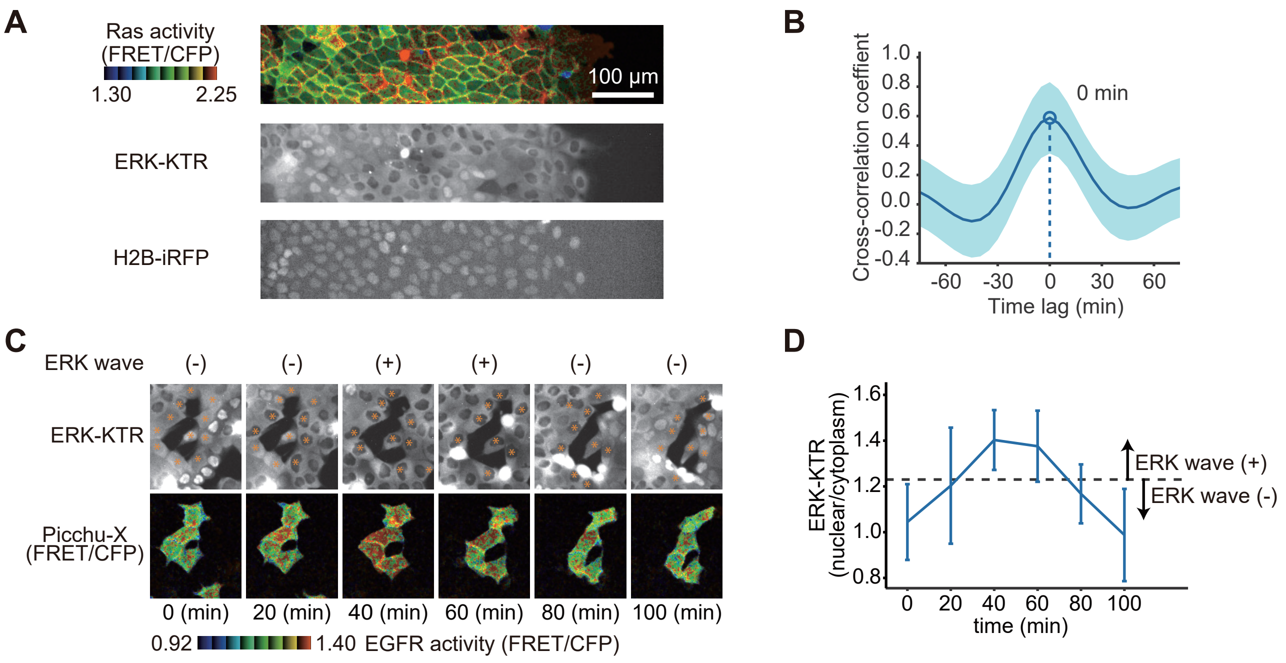

Fig. S2. Observation of ERK waves by ERK-KTR Related to Figure 1A and 1B. (**A**) MDCK cells co-expressing Raichu-Ras, ERK-KTR-mCherry, and H2B-iRFP were seeded on a glass-bottom dish and subjected to collective cell migration. Representative images were captured after 8 hours of migration. (**B**) Temporal cross-correlation between ERK activity and Ras activity. ERK activity was calculated by dividing mean intensity of H2B-iRFP by mean intensity of nuclear ERK-KTR. The blue line indicates the average temporal cross-correlation coefficients with standard deviations (SDs). n = 74 cells from an experiment. (**C**) Representative images of ERK wave analysis. (**D**) The mean ERK activities of neighboring cells are plotted with SD bars. A cell cluster was considered to be ERK wave (+) when the mean ERK activities were more than 1.23. This threshold was determined by averaging all of the ERK activity under the same medium conditions.

Journal of Cell Science • Supplementary information

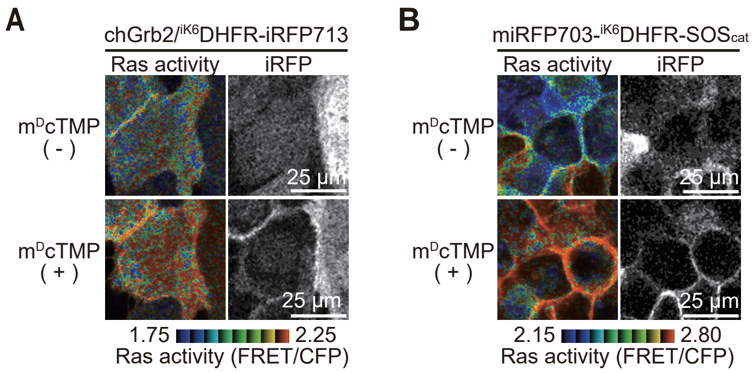

Fig. S3. SOS activation by SLIPT

Related to Figure 2D and 2G. MDCK cells expressing Raichu-Ras and chGrb2/^{iK6}DHFR-iRFP713 (**A**) or miRFP703-eDFHR-SOScat (**B**) were seeded on a glass-bottom dish to form a confluent epithelial sheet. Note that these cells are not migrating. Representative images of FRET/CFP and iRFP before and after the addition of 2 µM m^{D}cTMP are shown.

Journal of Cell Science • Supplementary information

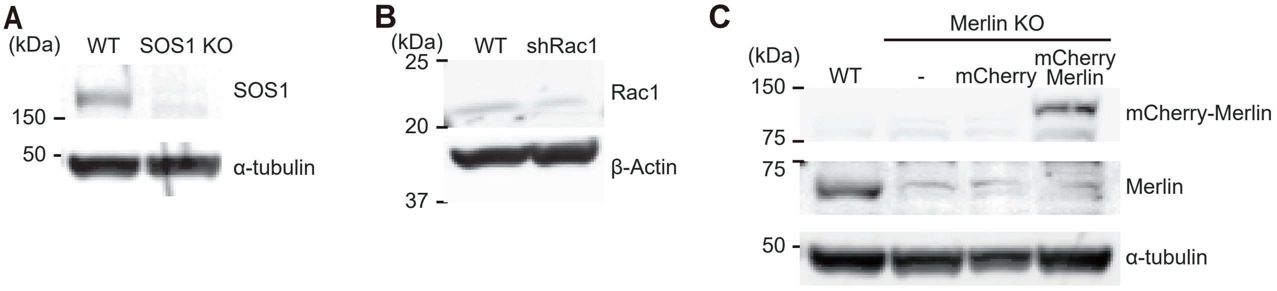

Fig. S4. Immunoblotting of SOS1, Rac1, and Merlin (**A-C**) Cell lysates were analyzed by immunoblotting with the indicated antibodies to assess knockout (KO), knockdown (KD), or re-expression.

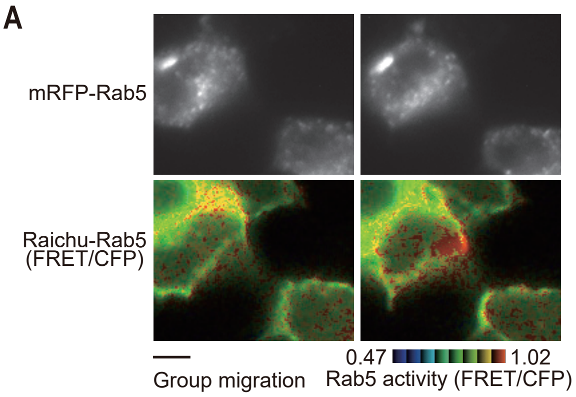

Fig. S5. Raichu-Rab5 fused to the carboxy terminus of Ki-Ras protein localizes to the plasma membrane

(**A**) Related to Figure 5A, 5B, and 5C. MDCK cells co-expressing Raichu-Rab5 and mRFP-Rab5 were mixed with the parental MDCK cells and subjected to collective cell migration.

Journal of Cell Science • Supplementary information

Table S1A. Plasmids in this paper.

Criteria	Plasmid name	AddGene#	Figure and movie	Source	Addgene# of source
Vector	pCMV-VSVG-RSV-Rev	N/A	N/A	Miyoshi et al., 1998	N/A
	psPAX2			A gift from Dr. Trono	12260
	pGP			Akagi et al., 2003	N/A
	pCMV-mPBase			A gift from Dr. Yusa<br>Yusa et al., 2009	
	pPBbleo-CAG-MCS				
	pPBpuro-CAG-MCS				
	pPBbsr2-CAG-MCS				
	pPBneo-UbC-MCS				
	pCSIIpuro-CAG-MCS			A gift from late Dr. Miyoshi	
	pPBpuro-miRFP703-eDHFR(69K6)-cRaf			Suzuki et al.,2022	209919
	pCAGGS-chGrb2/eDHFR(69K6)-iRFP713			Suzuki et al.,2022	178854
	lentiCRISPRv2			Sanjana et al., 2014	52961
	pX459			Ran et al., 2013	62988
ERK biosensor	pCSIIbsr-EKARrEV-NLS	173855	1C,1F,Movie1	Lin et al., 2021	173855
	pPBpuro-ERK-KTR-mCherry	214829	1J,1K,1L,1M,2A,2B,2D,2E,2G,2H,2I,2J,3A,3B,3C, 3D, 4C,4D,4E,5C,5D,S2A,S2B,S2C,S2D, Movie2,Movie4,Movie6	Regot et al., 2014	N/A
Tyrosine kinase biosensor	pPBbsr2-5102HRasCT(Picchu)	209917	1A,1D,1J,1K,S2C,S2D,Movie1,Movie2	Kurokawa et al, 2001	209917
Ras biosensor	pPBbsr2-Raichu-454HRasCT(Raichu-Ras)	176029	1B,1E,1G,1L,1M,2A,2B,2D,2E,2G,2H,2I,2J,3A, 3B,3C,3D,5E,S2A,S2B,S3A,S3B,Movie1	Hino et al., 2022	176029
Rac biosensor	pPBbsr2-RaichuEV-Rac1HRasCT	214818	4A,4B,4C,4D,4E,Movie3,Movie4	Komatsu et al., 2011	N/A
Rab5 biosensor	pCSIIbsr-Raichu-Rab5/PM	214819	5A,5B,5C,5D,S5A,Movie5,Movie6	kitano et al., 2008	N/A
SLIPT	pPBpuro-chGrb2/iK6DHFR-iRFP713	214828	2D,2E,S3A	Suzuki et al., 2022	178854
	pPBpuro-miRFP703-iK6DHFR-mSOS1-linkercat	214827	2G,2H,S3B	Suzuki et al., 2022, Lin et al.,2021	178849
dog Merlin-KO	lentiCRISPRv2-Merlin(dog)gRNA1	214823	3C,3D,4C,4D,4E,S4C	Boocock et al.,2020	N/A
	lentiCRISPRv2-bleo-Merlin(dog)gRNA2	214824	3C,3D,4C,4D,4E,S4C	Boocock et al.,2020	
dog SOS1-KO	pX459-SOS1(dog)-gRNA1	228755	2I,2J, 6A, 6B, 6D, 6E,S4A	Ran et al., 2013	62988
	pX459-SOS1(dog)-gRNA2	228756	2I,2J, 6A, 6B, 6D, 6E,S4B	Ran et al., 2013	
Rac1 KD	pSUPER-Rac1(dog)	228760	3A,3B,S4B	Hino et al, 2020	N/A
Dominant negative	pPBpuro-RasS17N	214820	5C,5D, 6A, 6B, 6D	Aoki et al., 2004	N/A
	pPBpuro-Rac1T17N	214821	3A,3B, 6A, 6B, 6D	Aoki et al., 2004	
	pPBpuro-Rab5S34N	214822	4C,4D, 6A, 6B, 6D	Imamura et al.,1998	
Nucleus marker	pCSIIbleo-H2B-iRFP	214830	1L, S2A, S2B	A gift from Dr. Miyanari	N/A
Rescue	pPBneo-UbC-mCherry-Merlin	228758	3C,3D,S4C	N/A	N/A
	pPBneo-UbC-mCherry	228759	3C,3D,S4C		
Endosome marker	pCNX2-mRFP-Rab5-WT	N/A	S5A	A gift from Dr. Miyanari	N/A

Journal of Cell Science • Supplementary information

Table S1B. Cell lines

Name	Original cell line	Source	Plasmid	Figure and movie	Additional notes
MDCK-5102HRasCT(Picchu)	MDCK	RIKEN BioResource Center	pPBbsr2-5102HRasCT(Picchu), pCMV-mPBase	1A, 1D, 1J, 1K, S2C, S2D, Movie1, Movie2	Single-cloned to omit the dim cells.
MDCK-454HRasCT(Raichu-Ras)	MDCK	RIKEN BioResource Center	pPBbsr2-Raichu-454HRasCT(Raichu-Ras), pCMV-mPBase	1B, 1E, 1G, 1L, 2A, 2B, 2I, 3A, 3C, 4E, Movie1, Movie2	Single-cloned to normalize the expression of Raichu-Ras.
MDCK-EKARrEV-NLS	MDCK	RIKEN BioResource Center	pCSIIbsr-EKARrEV-NLS, pCMV-VSV-G-RSV-Rev, psPAX2	1C, 1F, Movie1, 6A, 6B, 6D, 6E	Sorted to omit the cells expressing only CFP or YFP by recombination
MDCK-EKARrEV-NLS_RasS17N	MDCK-EKARrEV-NLS	RIKEN BioResource Center	pCSIIbsr-EKARrEV-NLS, pCMV-VSV-G-RSV-Rev, psPAX2, pPBpuro-RasS17N	1C, 1F, Movie1, 6A, 6B, 6D	Sorted to omit the cells expressing only CFP or YFP by recombination
MDCK-EKARrEV-NLS_Rac1T17N	MDCK-EKARrEV-NLS	RIKEN BioResource Center	pCSIIbsr-EKARrEV-NLS, pCMV-VSV-G-RSV-Rev, psPAX2, pPBpuro-Rac1T17N	1C, 1F, Movie1, 6A, 6B, 6D	Sorted to omit the cells expressing only CFP or YFP by recombination
MDCK-EKARrEV-NLS_Rab5S34N	MDCK-EKARrEV-NLS	RIKEN BioResource Center	pCSIIbsr-EKARrEV-NLS, pCMV-VSV-G-RSV-Rev, psPAX2, pPBpuro-Rab5S34N	1C, 1F, Movie1, 6A, 6B, 6D	Sorted to omit the cells expressing only CFP or YFP by recombination
MDCK SOS1KO_EKARrEV-NLS_RasS17N	MDCK	RIKEN BioResource Center	pX459-SOS1-1, pX459-SOS1-2, pCSIIbsr-EKARrEV-NLS, pCMV-VSV-G-RSV-Rev, psPAX2	1C, 1F, Movie1, 6A, 6B, 6D, 6E	Sorted to omit the cells expressing only CFP or YFP by recombination
MDCK-ERK-KTR-mCherry	MDCK	RIKEN BioResource Center	pPBpuro-ERK-KTR-mCherry, pCMV-mPBase	1J,1K,1L,1M, 2A, 2B, 2D, 2E, 2G, 2H, 2I, 2J, 3A, 3B, 3C, 3D, 4C, 4D, 4E, 5C, 5D, Movie2, Movie4, Movie6	Single-cloned to normalize the expression of ERK-KTR-mCherry.
MDCK-ERK-KTR-mCherry_H2B-iRFP_454HRas	MDCK	RIKEN BioResource Center	pPBpuro-ERK-KTR-mCherry, pCMV-mPBase,pCSIIbleo-H2B-iRFP, pCMV-VSV-G-RSV-Rev, psPAX2, pPBbsr2-Raichu454HRas	1M, S2A, S2B	Before transfection of pPBbsr2-Raichu-454HRas,single-cloned to normalize the expression of ERK-KTR-mCherry and H2B-iRFP.
MDCK-454HRasCT_chGrb2/eDHFR	MDCK-454HRasCT(Raichu-Ras)	This paper	pPBbsr2-Raichu-454HRasCT(Raichu-Ras), pCMV-mPBasepPBpuro-chGrb2/iK6DHFR-iRFP713	2D, 2E, S3A	Bulk
MDCK-454HRasCT_eDHFR-mSOS1-linkercat	MDCK-454HRasCT(Raichu-Ras)	This paper	pPBbsr2-Raichu-454HRasCT(Raichu-Ras), pCMV-mPBase,pPBpuro-miRFP703-iK6DHFR-mSOS1-linkercat	2G, 2H, S3B	Bulk
MDCK SOS1KO_454HRasCT	MDCK	RIKEN BioResource Center	pX459-SOS1-1, pX459-SOS1-2. pPBbsr2-Raichu-454HRasCT(Raichu-Ras), pCMV-mPBase	2I, 2J, S4A	Single-cloned to obtain SOS1KO cell
MDCK-454HRasCT_Rac1T17N	MDCK-454HRasCT(Raichu-Ras)	This paper	pPBbsr2-Raichu-454HRasCT(Raichu-Ras), pCMV-mPBase,pPBpuro-Rac1T17N	3A, 3B	Bulk
MDCK-454HRasCT_shRac1	MDCK-454HRasCT(Raichu-Ras)	This paper	pPBbsr2-Raichu-454HRasCT(Raichu-Ras), pCMV-mPBase,pSUPER-Rac1, pCMV-VSV-G-RSV-Rev, psPAX2	3A, 3B, S4B	Bulk
MDCK-454HRasCT MerlinKO	MDCK-454HRasCT(Raichu-Ras)	This paper	pPBbsr2-Raichu-454HRasCT(Raichu-Ras), pCMV-mPBase,lentiCRISPRv2-Merlin(dog)gRNA1, lentiCRISPRv2-bleo-Merlin(dog)gRNA2, pCMV-VSV-G-RSV-Rev, psPAX2	3C, 3D, 4E, S4C	Bulk
MDCK-454HRasCT MerlinKO_mCherry-Merlin	MDCK-454HRasCT MerlinKO	This paper	pPBbsr2-Raichu-454HRasCT(Raichu-Ras), pCMV-mPBase,lentiCRISPRv2-Merlin(dog)gRNA1, lentiCRISPRv2-bleo-Merlin(dog)gRNA2, pCMV-VSV-G-RSV-Rev, psPAX2, pPBneo-UbC-	3C, 3D, S4C	Sorted to achieve uniform expression level of mCherry-Merlin
MDCK-454HRasCT MerlinKO_mCherry	MDCK-454HRasCT MerlinKO	This paper	pPBbsr2-Raichu-454HRasCT(Raichu-Ras), pCMV-mPBase,lentiCRISPRv2-Merlin(dog)gRNA1, lentiCRISPRv2-bleo-Merlin(dog)gRNA2, pCMV-VSV-G-RSV-Rev, psPAX2, pPBneo-mCherry	3C, 3D, S4C	Sorted to achieve uniform expression level of mCherry
MDCK-RaichuEV-Rac1HRasCT	MDCK	RIKEN BioResource Center	pPBbsr2-RaichuEV-Rac1HRasCT, pCMV-mPBase	4A, 4B, 4C, 4D, 4E, Movie3, Movie4	Bulk
MDCK MerlinKO_RaichuEV-Rac1HRasCT	MDCK	RIKEN BioResource Center	lentiCRISPRv2-Merlin(dog)gRNA1, lentiCRISPRv2-bleo-Merlin(dog)gRNA2, pCMV-VSV-G-RSV-Rev, psPAX2, pPBbsr2-RaichuEV-Rac1HRasCT, pCMV-mPBase	4C, 4D, 4E	Bulk
MDCK-RaichuEV-Rac1HRasCT_Rab5S34N	MDCK-RaichuEV-Rac1HRasCT	This paper	pPBbsr2-RaichuEV-Rac1HRasCT, pCMV-mPBase, pPBpuro-Rab5S34N	4C, 4D, 4E	Bulk
MDCK-Raichu-Rab5/PM	MDCK	RIKEN BioResource Center	pCSIIbsr-Raichu-Rab5/PM, pCMV-mPBase	5A, 5B, 5C, 5D, S5A Movie5, Movie6	Sorted to omit the dim cells.
MDCK-Raichu-Rab5/PM_RasS17N	MDCK-Raichu-Rab5/PM	This paper	pCSIIbsr-Raichu-Rab5/PM, pCMV-mPBase,pPBpuro-HRasS17N	5C, 5D	Bulk

Journal of Cell Science • Supplementary information

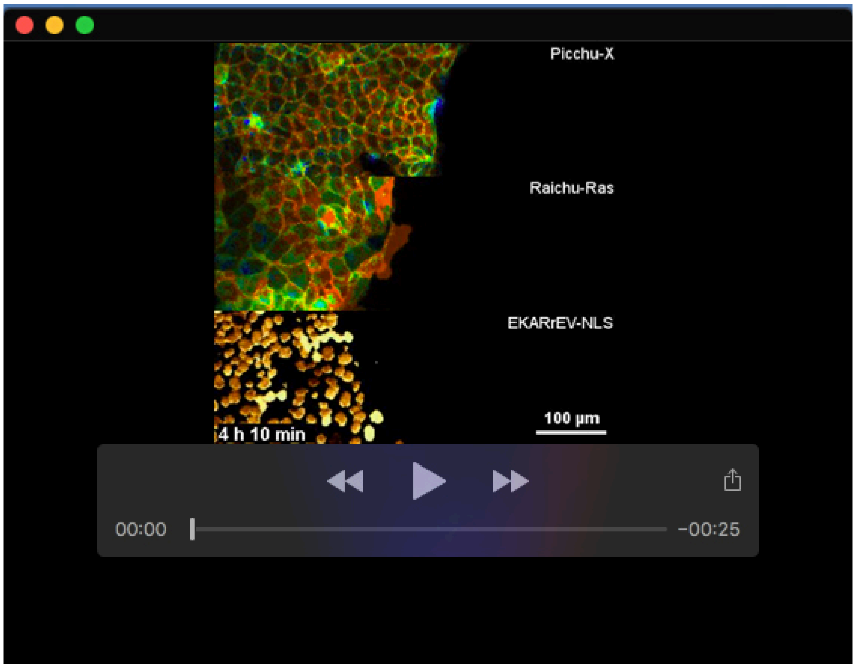

Movie 1. Activity of EGFR, Ras, and ERK during collective cell migration.

Related to Fig. 1A to 1C. MDCK cells expressing Picchu-X, Raichu-Ras, or EKARrEV were subjected to confinement release assay. The FRET/CFP ratio images show EGFR, Ras, and ERK activity. Time after the release of confinement is also shown. Scale bar, 100 µm.

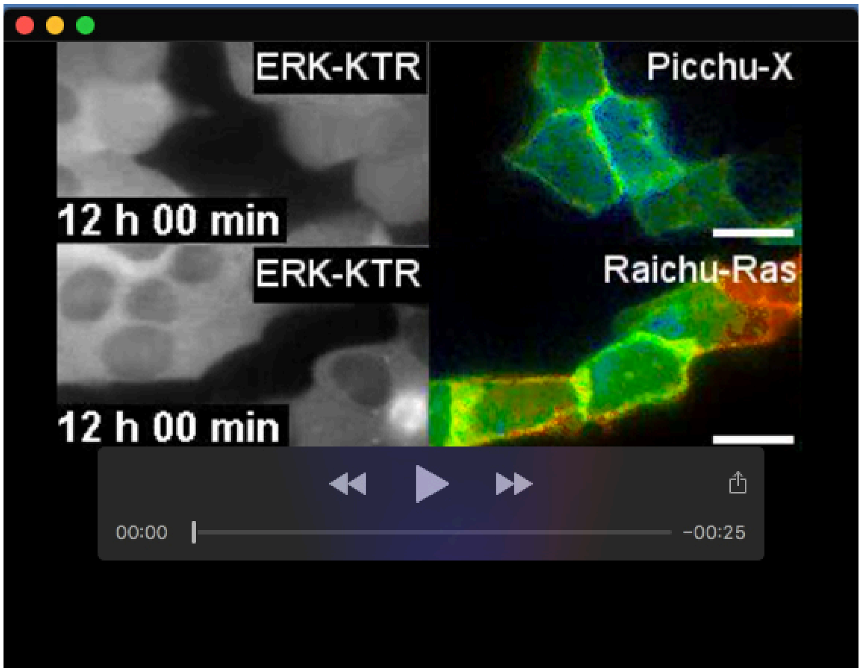

Movie 2. Subcellular activity of EGFR and Ras during collective cell migration.

Related to Fig. 1J and 1L. MDCK cells expressing Picchu-X or Raichu-Ras were mixed with those expressing ERK-KTR-mCherry, and subjected to confinement release assay. FRET/CFP ratio images show EGFR and Ras. Time after the release of confinement is also shown. Scale bar, 25 µm.

Journal of Cell Science • Supplementary information

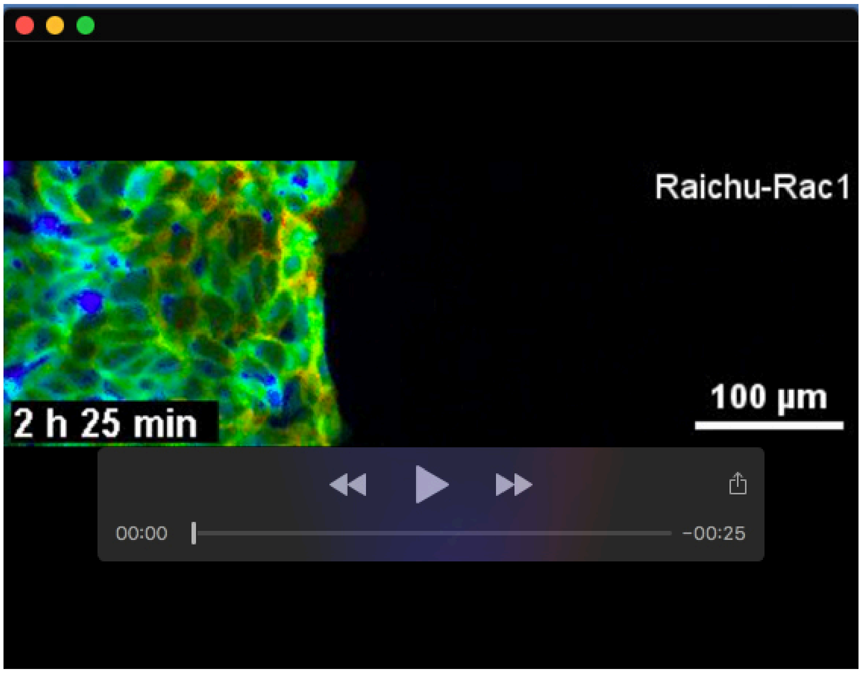

Movie 3. Rac1 activity during collective cell migration.

Related to Fig. 4A. MDCK cells expressing Raichu-Rac1 were subjected to confinement release assay. Time after the release of confinement is also shown. Scale bar, 100 µm.

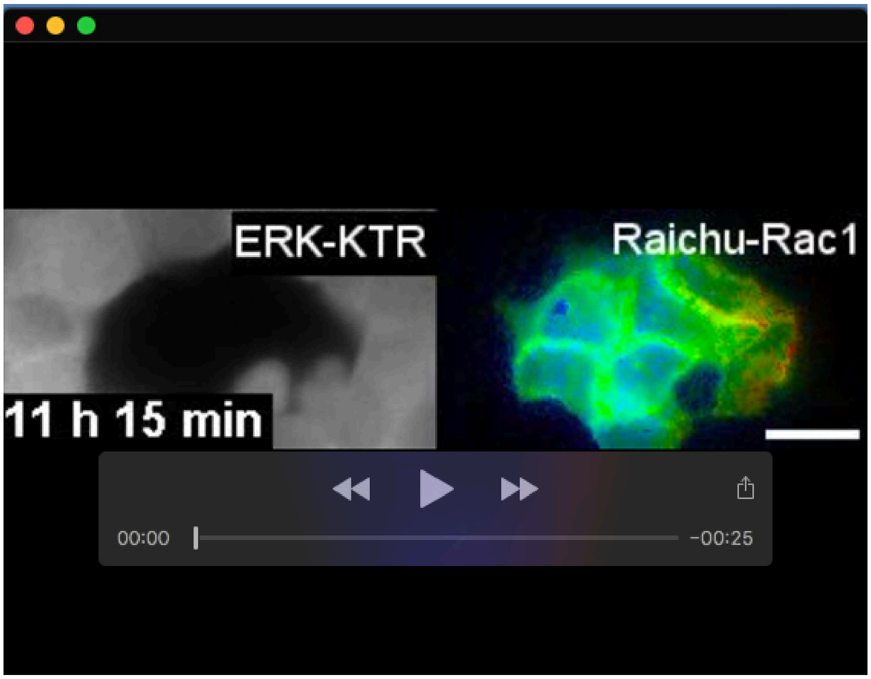

Movie 4. Subcellular activity of Rac during collective cell migration.

Related to Fig. 4C. MDCK cells expressing Raichu-Rac1 were mixed with those expressing ERK-KTR-mCherry, and subjected to confinement release assay. Time after the release of confinement is also shown. Scale bar, 25 µm.

Journal of Cell Science • Supplementary information

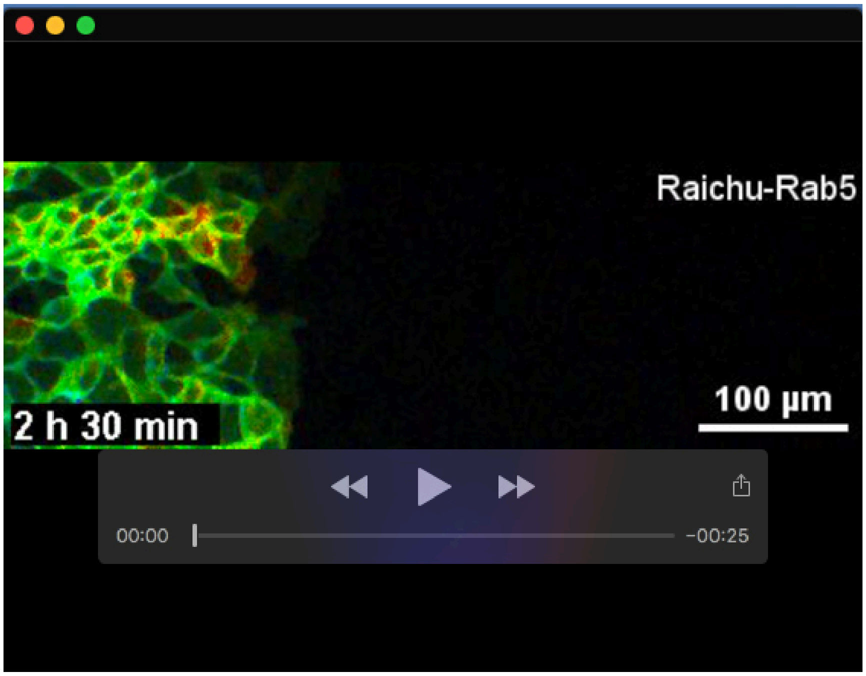

Movie 5. Rab5 activity during collective cell migration.

Related to Fig. 5A. MDCK cells expressing Raichu-Rab5 were subjected to confinement release assay. Time after the release of confinement is also shown. Scale bar, 100 µm.

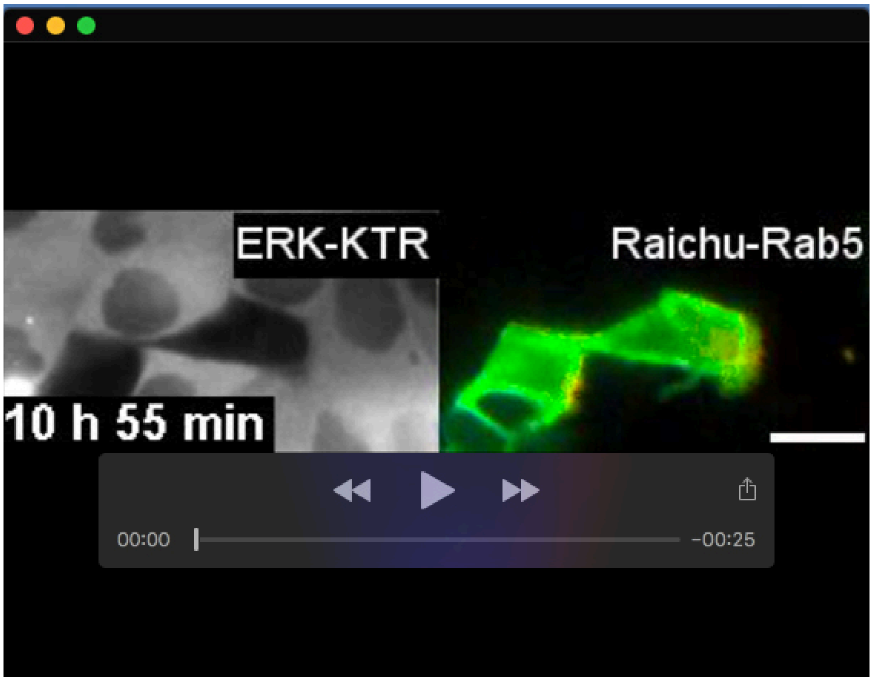

Movie 6. Subcellular activity of Rab5 during collective cell migration.

Related to Fig. 5C. MDCK cells expressing Raichu-Rab5 were mixed with those expressing ERK-KTR-mCherry, and subjected to confinement release assay. Time after the release of confinement is also shown. Scale bar, 25 µm.

Journal of Cell Science • Supplementary information
